# Potential Biological Impacts of Microplastics and Nanoplastics on Farm Animals: Global Perspectives with Insights from Bangladesh

**DOI:** 10.3390/ani15101394

**Published:** 2025-05-12

**Authors:** FNU Nahiduzzaman, Md Zaminur Rahman, Mst. Arjina Jannat Akhi, Mohammed Manik, Mst Minara Khatun, Md. Ariful Islam, Mohammad Nurul Matin, Md Azizul Haque

**Affiliations:** 1 Department of Microbiology and Hygiene, Bangladesh Agricultural University, Mymensingh 2202, Bangladesh; nahiduzzaman.2001055@bau.edu.bd (F.N.); zami_dvm@yahoo.com (M.Z.R.); mmkhatun@bau.edu.bd (M.M.K.); 2 Department of Electrical and Electronic Engineering, Eastern University, Dhaka 1205, Bangladesh; arjina.eu@yahoo.com; 3 Department of Environmental Science, Bangladesh Agricultural University, Mymensingh 2202, Bangladesh; mohammedmanik5012@gmail.com; 4 Department of Biotechnology, Yeungnam University, Gyeongsan 38541, Republic of Korea; nmatin2@yahoo.com; 5 Department of Genetic Engineering and Biotechnology, University of Rajshahi, Rajshahi 6205, Bangladesh

**Keywords:** contamination, environment, food safety, livestock, plastic pollution

## Abstract

Microplastics (MPs) and nanoplastics (NPs), formed through the degradation of larger plastic materials, are emerging pollutants of significant concern. While their impact on aquatic ecosystems is well documented, their effects on terrestrial, especially farm animals remain underexplored. This review assesses the potential threats of MPs and NPs to Bangladesh’s livestock sector by analyzing the results of experimental models and environmental studies. In Bangladesh, MPs and NPs have been detected in agricultural soils, air, water bodies, and aquatic organisms, indicating possible entry into animal systems through contaminated feed, water, and inhalation. Once internalized, these particles may trigger oxidative stress, inflammation, and tissue damage, impairing vital biological systems. Documented health consequences include reduced fertility, hematotoxicity, gut microbiota imbalance, gut–brain axis disruption, skeletal disorders, and metabolic dysfunction. Additionally, MPs and NPs can induce genomic changes, including altered gene expression and DNA hypomethylation, intensifying physiological damage and reducing productivity. Therefore, managing plastic contamination is vital in protecting animal health, ensuring food safety, and preserving human well-being around the globe, especially in vulnerable regions like Bangladesh. Given the critical role of livestock and poultry in ensuring food security and public health, the findings highlight an urgent need for comprehensive research and mitigation strategies.

## Simple Summary

Plastic pollution is not only a problem for oceans and rivers but also affects living organisms. Tiny plastic particles, called microplastics (MPs) and nanoplastics (NPs), can find their way into the food and water that farm animals consume. Once inside their bodies, these plastics can cause harm by putting stress on their organs and lowering the quality of the meat, milk, and eggs they produce. Even more concerning, these plastics can remain in the animals’ tissues, creating several pathophysiological complexities. To protect both animals and humans, scientists need to understand how widespread this problem is and develop ways to prevent it.

## 1. Introduction

Farm animals, encompassing both livestock and poultry, are integral to global agricultural systems, playing a vital role in ensuring food security and supporting economic development. Livestock includes domesticated species such as cattle, sheep, goats, pigs, and horses, while poultry comprises birds such as chickens, ducks, turkeys, and pheasants [1,2]. In 2020, global meat consumption was approximately 360 million tons, and it is projected to increase by 14% by 2030 [3]. In Bangladesh, these sectors are equally vital, contributing 1.8% to national GDP and sustaining rural livelihoods for millions through employment and nutrition [4,5]. However, the rapid expansion of these industries coincides with escalating environmental threats, particularly from plastic pollution, which jeopardizes animal health, food safety, and economic stability on both global and local scales.

Plastics, derived predominantly from petrochemicals, have become ubiquitous in modern society since the invention of Bakelite in the early 20th century [6]. Global plastic production surged from 1.7 million tons in the 1950s to over 367 million tons in 2020, with mismanaged waste now exceeding 218 million tons annually [7,8]. This deluge of plastic debris degrades into microplastics (MPs, <5 mm) and nanoplastics (NPs, <100 nm) through environmental processes like UV radiation, mechanical abrasion, and microbial activity [9,10]. These particles infiltrate terrestrial and aquatic ecosystems globally, contaminating soil, water, and air [11].

In Bangladesh, plastic pollution is equally alarming: the country produces 1.2 million metric tons of plastic annually, with 800,000 tons improperly discarded into rivers, farmland, and urban areas due to inadequate waste management infrastructure [12,13]. Monsoon floods exacerbate this issue by dispersing plastic waste into grazing fields and waterways, accelerating its fragmentation into MPs/NPs [14]. Recent studies have confirmed the presence of microplastics in various environmental compartments across Bangladesh, including surface water, sediments, and even atmospheric fallout. For instance, microplastics have been detected in river systems such as the Buriganga, Meghna, and Karnaphuli, with concentrations ranging from hundreds to thousands of particles per cubic meter in water and per kilogram in sediments, highlighting widespread environmental contamination and the growing risk of trophic transfer into terrestrial food chains [15,16].

Globally, MPs/NPs have been detected in livestock and poultry feeds and products, including meat, milk, eggs, and manure, raising concerns about human exposure through the food chain [17,18,19]. These particles enter the animals via the ingestion of contaminated feed and water, inhalation of airborne NPs, and dermal contact with plastic-laden environments [20,21]. Once internalized, MPs/NPs induce oxidative stress, endocrine disruption, and immune dysfunction in livestock [22].

The authors of a study conducted in Germany found that exposure to 0.3 μm and 1.1 μm polystyrene microplastics for 24 h significantly reduced oocyte maturation to 42.0% and 41.0%, respectively, compared to 68.1% in the control group [23]. In Bangladesh, the authors of another study found 550 to 11,600 microplastic particles/kg of fish feed [24], providing strong evidence of the potential presence of plastic particles in poultry and livestock feed as well. Despite global advancements in understanding MP/NP risks, Bangladesh’s livestock sector remains critically understudied. Moreover, Bangladesh lacks comparable regulations, and most people remain almost unaware of plastic contamination risks [25,26].

Research in Bangladesh has focused narrowly on aquatic ecosystems. Several studies have reported the presence of MPs in fish of both river and ocean origin [27,28,29], while terrestrial livestock systems, central to food security, are overlooked. No national studies have assessed MPs/NPs in dairy products or meat, despite global evidence of their transfer to humans. This disparity highlights an urgent need to contextualize global findings within Bangladesh’s socioecological landscape, where smallholder farming, climate vulnerabilities, and informal waste practices amplify exposure risks.

This review synthesizes global evidence on MP and NP contamination in farm animals and assesses its potential implications for Bangladesh. Although no studies have yet reported the presence of MPs/NPs in farm animals, feed, or animal-derived products in Bangladesh, the documented environmental contamination suggests a possible risk. Drawing on the findings and experimental model studies, this review evaluates the biological impacts of MP/NP exposure and underscores the need for context-specific policies to protect animal health, ensure food safety, and support agricultural sustainability throughout the world, especially in Bangladesh.

## 2. Plastic Use and Waste Management Practices in Bangladesh

Plastic is deeply integrated into the daily lives of people in Bangladesh, with its use spanning across various sectors and activities. Commonly used materials include plastic bags, packaging materials, bottles, household items, toys, and electronic products [14]. The widespread availability, user-friendliness, and low cost of plastic products have made them a preferred choice for consumers and businesses. In urban areas, disposable plastic items such as food containers, cutlery, and cups are prevalent, especially in the fast-food industry and street vending [30]. The agricultural sector also relies on plastics, particularly in the form of mulch films, irrigation pipes, and greenhouse covers, which aid in crop production [31,32]. In the healthcare sector, plastics are essential for producing medical supplies such as syringes, gloves, and IV bags [33]. The COVID-19 pandemic has exacerbated this phenomenon, doubled plastic waste generation, and increased single-use plastic usage [34]. The textile and garment industry, a significant contributor to the economy, utilizes plastics in the form of synthetic fibers like polyester [35]. Plastics are also used in construction, with materials like PVC pipes and plastic roofing sheets becoming common. Bangladesh is an overpopulated country with numerous forms of environmental mismanagement. Plastic pollution has become a severe one-health issue for the country [14]. This country faces a significant challenge in managing plastic waste, with improper disposal and inadequate recycling infrastructure exacerbating the issue [13,36]. Bangladesh’s plastic waste generation is substantial, with per capita waste generation at 7.9 kg annually [37]. The plastic waste management policy of the country is not fully followed, both in cities and the countryside [38]. Consequently, huge amounts of plastic waste are accumulating in agricultural lands, ponds, lakes, rivers, and seabeds [13].

## 3. Routes of MP and NP Infiltration into Farm Animals

**Figure 1:**
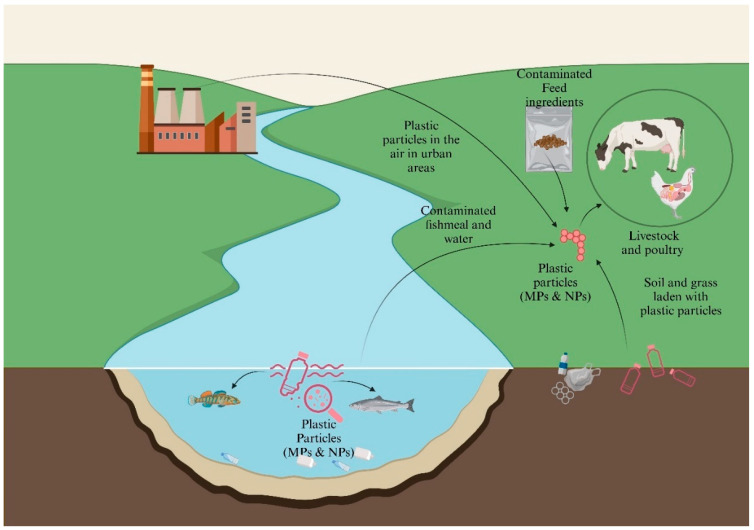
Pathways of microplastic and nanoplastic entry into livestock and poultry systems.

MP-NP pollution has become a growing concern for livestock and poultry globally, as plastic particles have been detected in a variety of animal species and environments. Studies have shown that these small plastic particles are present in feed, water sources, and even in the air, exposing animals to continuous ingestion and inhalation of plastic contaminants [39,40]. Once ingested, MPs can accumulate in the gastrointestinal tract of animals, leading to potential health issues such as inflammation, reduced nutrient absorption, and physical damage to internal organs [41]. Research in several countries has revealed the presence of MPs in livestock, including cattle, sheep, and poultry [42]. Contaminated feed and water sources are the primary routes through which plastics accumulate from polluted environments [20,21]. Fish meal, meat and bone meal (MBM), and soybean meal are important protein sources for livestock and poultry feeds in Bangladesh [43]. Several studies conducted in Spain, Ireland, China, and India reported that almost every investigated animal and poultry feed, including fishmeal and soybean meal, was contaminated with MPs such as polypropylene, polystyrene, polyethylene, polyamide, polyethylene terephthalate, and Polyvinyl Chloride MPs [40,44,45,46]. Manure and sewage sludge, often used as fertilizers, can introduce MPs into the soil, which are then ingested by animals. Animals grazing on plastic-laden soils or ingesting contaminated forage are also at risk of plastic particles [47]. Additionally, MPs-NPs can enter through the inhalation of airborne particles, particularly in industrial or urban areas [48]. The use of plastic-containing products, such as packaging or feed additives, further exacerbates exposure [20,47]. The infiltration pathways are shown in Figure 1.

## 4. Types of Plastic Particles in the Poultry and Livestock Sectors

MP-NP pollution poses significant risks to the poultry and livestock industries due to contamination in feed, water, and the environment. Various studies have demonstrated the widespread presence of MPs in animal tissues, revealing the extent of this pollution. In South Caloocan, Philippines, MPs, including fragments, pellets, and films made of polyethylene (PE), polypropylene (PP), and polystyrene (PS), were identified in the gizzards and intestines of chickens, highlighting exposure from polluted feed and the environment [49]. In Mexico, larger plastic particles composed of PET were found in the crops of chickens, while smaller MPs (<5 mm) accumulated in the gizzards, primarily due to soil and feed ingestion [50]. Similarly, in Indonesia, the intestines of ducks were contaminated with MPs, primarily fragments and filaments composed of polymers such as nylon (PA), PET, PVC, and poly(n-butyl methacrylate) (PBM) [51]. Furthermore, polystyrene (PS) MPs induced oxidative stress and inflammation in chicken testicular tissue [52]. A study reported significant organ damage and reduced growth rates in poultry exposed to MPs through feed. Beyond poultry, MPs composed of PE, polystyrene (PS), and PVC have been detected in livestock’s meat, milk, and blood, raising serious concerns about their transfer through the food chain to humans [53]. The pervasive presence of MPs in animal tissues, as shown in Table 1, underscores the need for global monitoring, sustainable agricultural practices, and regulatory measures to mitigate the impact of MP pollution on animal health and food safety.

## 5. Potential Effects of MPs and NPs on Farm Animals

**Figure 2:**
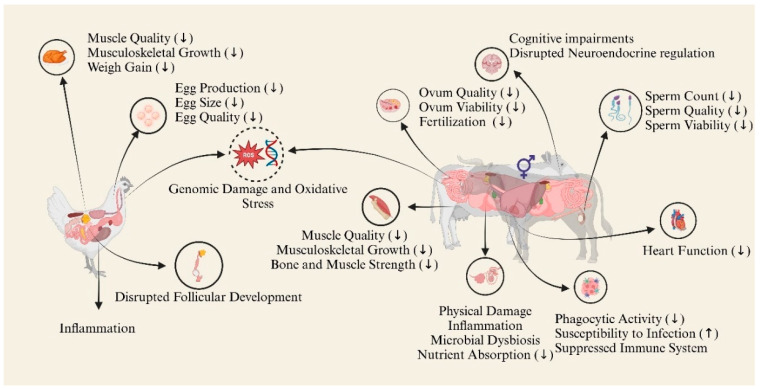
The adverse effects of MPs-NPs on different biological systems of livestock and poultry; (↑ represent the increased and ↓ represent the decreased).

MPs-NPs have become pervasive environmental pollutants, raising significant concerns for livestock and poultry health. While direct studies on these animals are limited, insights from several experimental models provide a basis for understanding potential impacts. Studies on rodents and aquatic species demonstrate that MPs and/or NPs induce oxidative stress, inflammation, immune system dysfunction, and reproductive toxicity, which could similarly affect farm animals, including livestock and poultry. Disrupted gut microbiota, impaired nutrient absorption, and reduced fertility observed in models (Table 2) suggest adverse effects on growth, productivity, and overall health in farm animals, compromising meat and egg quality while posing food safety risks due to the bioaccumulation of plastic particles. The adverse effects of MPs and NPs on the different biological systems of farm animals are demonstrated in Figure 2. Moreover, their adverse effects on the various biological systems of farm animals are extensively discussed herein.

### 5.1. Adverse Effects on the Reproductive System

The presence of MPs-NPs in the environment has raised significant concerns regarding their potential impacts on reproductive health in farm animals. Studies conducted on various experimental models suggest that exposure to MPs and NPs can lead to reproductive toxicity through mechanisms such as oxidative stress, endocrine disruption, inflammatory responses, genetic alterations, and direct cellular damage, which could have similar implications for livestock and poultry species.

Research on rodents has shown that polystyrene microplastics (PS-MPs) can accumulate in reproductive tissues, leading to ovarian inflammation, reduced oocyte quality, and altered biomarker levels such as interleukin-6 (IL-6), glutathione (GSH), and mitochondrial dysfunction [57]. MPs have been found to impair oocyte maturation and reduce the first polar body extrusion rate, ultimately affecting female fertility [57]. The disruption of mitochondrial membrane potential and endoplasmic reticulum calcium homeostasis further compromises oocyte viability, which may translate into decreased reproductive efficiency in livestock. MPs also disrupt ovarian folliculogenesis by interfering with hormonal signaling pathways essential for follicle development [77] and adsorb endocrine-disrupting chemicals (EDCs) such as bisphenol A (BPA), which mimic or antagonize natural hormones, thereby affecting ovulation and corpus luteum formation [78]. These results suggest the potential effects on poultry species in decreasing egg production.

Moreover, nanoplastics, due to their smaller size, can penetrate deeper into tissues and cross critical barriers such as the placental barrier, leading to oxidative stress and inflammation in placental tissues, which impairs fetal development and increases the risk of low birth weight and neonatal mortality [79].

Male reproductive health is equally at risk. Studies in experimental models have demonstrated reduced sperm motility, increased sperm deformities, and significant decreases in testosterone levels following exposure to PS-MPs [58,77]. MPs can activate oxidative stress pathways, particularly the p38 MAPK and JNK signaling cascades, leading to increased reactive oxygen species (ROS) generation and sperm apoptosis [58]. Additionally, MPs can cross the blood–testis barrier, inducing testicular inflammation and impairing spermatogenesis, which may result in long-term fertility issues in male animals [62]. MPs have been demonstrated to penetrate the blood–testis barrier and accumulate in testicular tissues, causing histopathological changes such as seminiferous tubule damage, multinucleated monocytes, and disorganized spermatogenic cells [65]. These changes indicate that farm animals consuming MP-contaminated feed or water may experience similar reproductive impairments, reducing breeding success and herd productivity. MPs also absorb persistent organic pollutants (POPs), which compound reproductive toxicity by altering the hypothalamic–pituitary–gonadal axis and reducing levels of key reproductive hormones such as testosterone and estradiol [80].

The toxicokinetics of MPs suggest a high potential for transgenerational reproductive effects. Research has shown that MP exposure during gestation in rodent models results in developmental abnormalities in offspring, including reduced birth weights, altered steroid hormone levels, and delayed gonadal maturation [74,79]. Further evidence from aquatic models underscores these risks. Zebrafish studies have shown that MPs disrupt gonadal development, reduce fecundity, and impair endocrine signaling, suggesting similar outcomes for livestock and poultry species exposed to contaminated water or feed [81]. Transgenerational toxic effects may also involve epigenetic modifications that could persist across generations, further exacerbating reproductive challenges in livestock populations.

Endocrine disruption is another major concern, as MPs have been found to interfere with the hypothalamic–pituitary–gonadal (HPG) axis, resulting in altered steroidogenesis, imbalanced sex hormone levels, and disrupted reproductive cycles [82]. Changes in testosterone and estradiol levels may impact reproductive timing, ovulation rates, and overall fertility in farm animals. Additionally, transcriptomic analyses have revealed that MP exposure can downregulate genes involved in steroid biosynthesis, reproductive signaling pathways, and cellular homeostasis [71]. These molecular disruptions may contribute to reduced gamete viability, embryonic developmental failures, and altered reproductive organ function in exposed animals.

**Figure 3:**
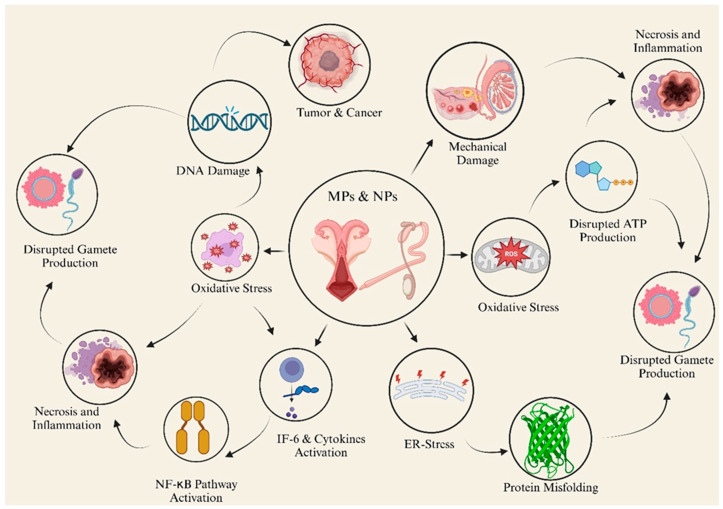
The mechanism of the reproductive toxicity of MP-NPS.

Farm animals are particularly vulnerable to MP exposure due to their continuous ingestion of contaminated pasture, fodder, concentrate, and water. MPs have been detected in soil, water, and feed, raising concerns about chronic exposure and cumulative reproductive health effects [63]. Once ingested, MPs accumulate in the gastrointestinal tract and translocate to reproductive organs through the systemic circulation [83]. Over time, chronic exposure to MPs may lead to cumulative damage to reproductive tissues with significant implications for livestock productivity and economic sustainability, as shown in Figure 3 [84].

Addressing these risks requires comprehensive waste management practices, the development of MP-free feed sources, and targeted research to mitigate the long-term impacts of MPs and NPs on animal reproduction [85,86]. While the existing evidence is derived primarily from experimental studies, the potential implications for farm animals are concerning. If MPs exert similar reproductive toxicities in livestock and poultry, they could lead to decreased fertility, disrupted reproductive cycles, and compromised offspring health, ultimately affecting agricultural productivity. Future research focused on species-specific studies is essential to fully understand and mitigate these risks in animal husbandry.

### 5.2. Nervous and Immune Systems

Microplastics and nanoplastics pose a significant threat to the immune system in various organisms, including livestock, poultry, and other species. These synthetic particles induce chronic inflammation and immune dysregulation by triggering the release of pro-inflammatory cytokines such as interleukin-6 (IL-6) and tumor necrosis factor-alpha (TNF-α), thereby weakening immune defenses [87]. In rodents, MPs were found to disrupt hematopoietic functions in the spleen and bone marrow, reducing resistance to infections by altering immune populations and impairing phagocytic activity [72]. Similarly, MPs destabilized the gut microbiome in mice, decreasing beneficial bacteria such as *Bifidobacterium* while promoting pro-inflammatory species, ultimately compromising gut-associated lymphoid tissue (GALT) and mucosal immunity [81]. These disruptions highlight the systemic impact of MPs-NPs on immune homeostasis, with potential consequences for farm animal health and productivity.

In livestock and poultry, MPs-NPs exposure compromises immune cell functions, particularly phagocytosis, the process by which macrophages and other immune cells clear pathogens, leading to a compromised immune response and increased susceptibility to infections [88]. Prolonged MPs-NPs exposure results in chronic immune activation, characterized by elevated levels of immune cells such as neutrophils and macrophages, which further amplify inflammatory responses through the release of cytokines like IL-1β and TNF-α [89]. Additionally, MPs-NPs reduce the expression of anti-inflammatory cytokines such as IL-10, leading to unchecked inflammation, tissue damage, and impaired recovery from infections [57].

Moreover, MPs-NPs exposure significantly impacts the gastrointestinal immune environment by disrupting gut microbiota and compromising the gut barrier, which serves as the first line of defense against ingested pathogens [67]. This increased gut permeability allows harmful substances and pathogens to enter the bloodstream, exacerbating systemic inflammation and immune dysfunction in farm animals [90]. Such chronic inflammation not only leads to organ damage but also increases the risk of autoimmune conditions, which could severely impair productivity and overall health [91]. Additionally, MPs act as vectors for toxic substances, including polycyclic aromatic hydrocarbons (PAHs), which exacerbate immune dysregulation and reduce disease resistance, as demonstrated in marine organisms exposed to contaminated MPs [92].

The immunotoxic effects of MPs-NPs highlight the urgent need for further research into their long-term impacts on farm animal health. The immune dysregulation caused by MP-NP exposure not only affects disease resistance but also contributes to chronic inflammatory conditions, increasing the risk of autoimmune disorders and organ damage in animals. These adverse effects highlight the urgent need for further research and mitigation strategies to prevent long-term health and productivity losses in the livestock and poultry industry.

### 5.3. Adverse Effects on the Digestive System and Metabolism

MPs-NPs have emerged as contaminants of concern in terrestrial environments, with growing evidence suggesting their detrimental effects on the digestive system and metabolism of mammals or other animals. In farm animals, ingestion of MPs and NPs can lead to significant disruptions in gastrointestinal function, nutrient absorption, microbiota composition, and metabolic homeostasis. While most studies have focused on rodent and avian models, the observed toxicological effects provide valuable insights into the potential risks faced by livestock animals exposed to these persistent pollutants [66,81].

MPs have been shown to impair the integrity of the intestinal barrier, making animals more susceptible to inflammation, increased permeability, and reduced mucus production. Studies have demonstrated that MPs can lead to decreased mucus secretion and damage to the intestinal epithelial lining, resulting in compromised gut barrier function and increased exposure to harmful pathogens [66]. Additionally, MPs alter the expression of genes responsible for maintaining intestinal tight junctions, further exacerbating intestinal permeability and promoting inflammatory responses [93]. Polystyrene MPs have been linked to the necrosis and pyroptosis of intestinal tissues in avian models, accompanied by disruptions in the gut vascular barrier, allowing toxins to enter the systemic circulation [68]. These effects can be significantly observed in poultry species, potentially leading to compromised intestinal integrity, increased susceptibility to systemic infections, impaired nutrient absorption, and overall reduced health and productivity.

A growing body of research indicates that MPs significantly alter the gut microbiota, leading to dysbiosis and associated metabolic disorders. Exposure to MPs has been linked to a decrease in beneficial gut bacteria, such as Firmicutes and Actinobacteria, while increasing harmful bacterial populations, including Proteobacteria [67,94]. Such microbial imbalances can lead to inflammatory responses, impaired digestion, and disturbances in energy metabolism. Alterations in the gut microbiota have also been linked to disruptions in bile acid metabolism, lipid digestion, amino acid metabolism, and short-chain fatty acid production, all of which play critical roles in maintaining overall metabolic homeostasis [66,69]. The metabolic consequences are particularly concerning, as farm animals exposed to MPs may suffer from malnutrition, weakened immune defenses, and reduced productivity, with cascading effects on agricultural yields and food safety.

Beyond the gut, MPs and NPs have been found to impact systemic metabolism by inducing oxidative stress and mitochondrial dysfunction in key metabolic organs. Research has shown that MPs accumulate in the liver, where they cause oxidative damage, lipid accumulation, and disruptions in hepatic metabolism [95,96]. Liver function is critical for nutrient metabolism, toxin detoxification, and overall energy balance in terrestrial animals; therefore, any impairment due to MP exposure could have widespread consequences for animal health and productivity. Additionally, MPs have been found to induce changes in glucose and lipid metabolism, increasing the risk of metabolic disorders akin to those observed in high-fat diet-fed rodents exposed to MPs [64]. The observed metabolic disruptions include alterations in insulin signaling, lipid accumulation, and increased inflammatory markers, all of which could contribute to metabolic diseases such as fatty liver syndrome and insulin resistance in animals.

MP exposure has also been shown to influence gut–liver interactions, a key axis in metabolic regulation. Studies indicate that MPs can translocate from the gut into the systemic circulation, where they trigger immune responses and promote metabolic inflammation [68]. MPs have been linked to increased levels of lipopolysaccharides (LPS), a major component of bacterial cell walls that contributes to chronic low-grade inflammation when it enters circulation [69]. This inflammation can, in turn, disrupt liver metabolism and contribute to conditions such as non-alcoholic fatty liver disease (NAFLD), which is increasingly recognized as a concern in both human and veterinary medicine [64].

Additionally, MPs impair metabolic function in other organs, including skeletal muscle and adipose tissue. Studies on rodent models have demonstrated that MPs can delay muscle regeneration, alter muscle fiber composition, and promote lipid deposition in muscle tissue, thereby affecting overall muscle growth and performance [61]. These findings are particularly relevant for livestock and poultry species raised for meat production, as any disruptions in muscle growth and metabolic efficiency could directly impact meat quality and economic outcomes.

Furthermore, MPs and NPs affect energy metabolism at the cellular level by interfering with mitochondrial function. Research has shown that MPs induce oxidative stress, leading to increased reactive oxygen species (ROS) production, mitochondrial membrane damage, and disruptions in ATP production [95,97]. Mitochondria play a crucial role in energy metabolism, and their impairment can result in reduced feeding efficiency, slower growth rates, and increased susceptibility to metabolic disorders in livestock and poultry species. The disruption of mitochondrial function has also been linked to increased adipogenesis, whereby MPs promote the conversion of myoblasts into adipocytes, potentially altering body composition and fat deposition in animals [61].

**Figure 4:**
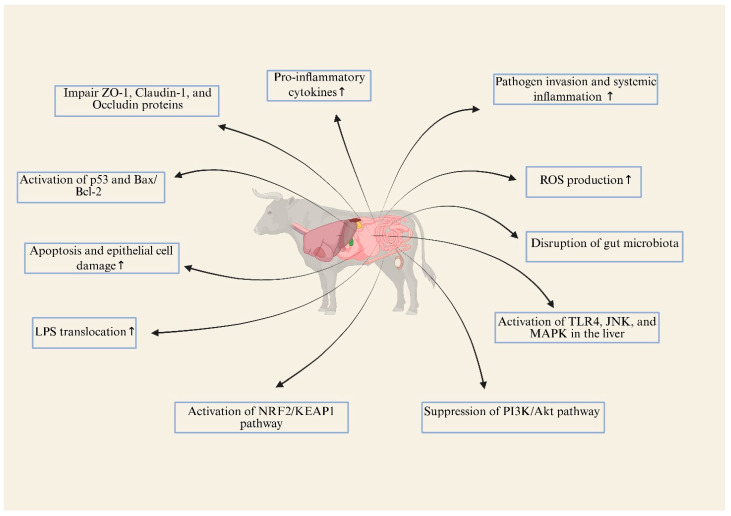
Adverse effects of MPs-NPs on the digestive system; (↑ represent increased).

MPs also act as carriers for toxic pollutants and heavy metals, which can exacerbate their harmful effects by leaching toxic substances into tissues upon ingestion [98]. Additionally, MPs interfere with endocrine regulation, acting as endocrine disruptors by altering hormone signaling pathways involved in appetite regulation, energy homeostasis, and lipid metabolism [64]. Disruptions in these pathways (Figure 4) may contribute to abnormal weight gain, impaired reproductive function, and metabolic inefficiencies in farm animals.

Agricultural animals are continuously exposed to MPs through contaminated water, feed, and environmental pollution, making it essential to assess the risks posed by prolonged ingestion of these particles. MPs and NPs pose a significant threat to the digestive health and metabolic function of livestock, with potential consequences for animal growth, productivity, and overall well-being. Their ability to alter gut microbiota, impair intestinal barrier function, induce oxidative stress, and disrupt metabolic pathways underscores the urgent need for further research into their effects on livestock species. As plastic pollution continues to increase, addressing the potential risks associated with MPs in agricultural settings will be crucial for ensuring the health and sustainability of farm animals.

### 5.4. Genomic Effects

Microplastics and nanoplastics are emerging environmental pollutants with profound implications for genomic stability and breed quality. Research has demonstrated that MPs and NPs infiltrate biological systems, accumulate in critical organs, and induce genetic and epigenetic modifications, leading to genomic instability [99]. Their ability to penetrate biological barriers such as the blood–brain barrier and reproductive tissues has been linked to neurological damage, decreased oocyte quality, inflammation, and reduced sperm motility due to increased oxidative stress [57,62].

One of the primary mechanisms of genomic degradation is oxidative stress, which results in the overproduction of reactive oxygen species (ROS), causing DNA damage, lipid peroxidation, and mitochondrial dysfunction [97]. The prolonged accumulation of ROS leads to the breakdown of cellular integrity, resulting in apoptosis and autophagy dysregulation [71]. In zebrafish models, MPs have been shown to alter gene expression associated with oxidative stress and apoptosis, thereby compromising DNA repair mechanisms and initiating various cellular responses [100,101]. Additionally, MPs induce heritable genetic alterations, as zebrafish offspring of exposed parents exhibited reduced hatching success and deformities, emphasizing the long-term threat to species viability [101].

In mammalian models, polystyrene MPs caused double-strand DNA breaks, impaired repair pathways, and dysregulated inflammatory gene expression, further contributing to genomic instability [102]. Hepatic tissues exhibited disrupted transcription of genes involved in lipid and carbohydrate metabolism, suggesting systemic genomic instability [95]. In vitro experiments using human cell lines like Caco-2 and A549 demonstrated that MPs disrupt the cell cycle, leading to chromosomal abnormalities and micronuclei formation [103]. This highlights the potential for MPs to cause widespread genomic damage across multiple species, including humans.

A study reported that early-life exposure to polystyrene microplastics (PSMPs) in zebrafish led to DNA hypomethylation upon re-exposure, indicating potential long-term epigenetic effects [104]. Transcriptomic analyses of MP-exposed organisms revealed significant changes in gene expression related to inflammation, metabolism, and circadian regulation, suggesting that these particles interfere with epigenetic regulatory pathways [63]. Prolonged exposure to MPs increases the likelihood of mutations and cellular dysfunction, further compromising genomic integrity.

MPs act as carriers of toxic chemicals such as polycyclic aromatic hydrocarbons (PAHs), which amplify genotoxic effects and increase DNA strand breaks [92]. Studies in lugworms demonstrated that PAH-adsorbed MPs heightened micronuclei formation and DNA fragmentation, exacerbating genetic instability. This phenomenon highlights the dual threat of MPs as both physical pollutants and chemical vectors, adding a layer of genomic degradation risk.

Moreover, MPs disrupt the hypothalamic–pituitary–gonadal (HPG) axis, affecting reproductive health, decreasing fertility, and causing transgenerational abnormalities [82]. In marine medaka, MP exposure led to significant downregulation of genes in the HPG axis, impairing reproductive function and reducing offspring viability [82]. In rodent models, MPs interfered with testosterone secretion and sperm function by activating the p38 MAPK and JNK pathways, further contributing to reproductive toxicity [58]. Additionally, MPs disturb the gut microbiota–hypoxanthine–Wnt axis, which affects hematopoietic stem cell self-renewal and immune system regulation [73]. These disruptions heighten susceptibility to infections and metabolic disorders, further degrading breed quality and resilience.

The continuous presence of MPs in tissues such as the liver, brain, and reproductive organs exacerbates genomic instability by overwhelming nucleotide excision repair (NER) and base excision repair (BER) pathways, thereby increasing susceptibility to mutations, cancer, and neurodegenerative disorders [79]. In mice models, MPs and a high-fat diet promoted oxidative phosphorylation and chemical carcinogenesis pathways in renal cells, emphasizing the role of MPs in tumorigenesis [96]. Furthermore, MPs disrupt cholesterol biosynthesis and DNA replication in hepatic cells, increasing the likelihood of metabolic dysregulation and carcinogenesis [105].

Collectively, the cumulative genomic degradation caused by MPs and NPs significantly affects breed quality, leading to reduced reproductive success, impaired development, and increased disease susceptibility. Their ability to alter gene expression, induce mutations, and disrupt critical physiological pathways jeopardize genetic fidelity across generations. This raises substantial concerns for biodiversity conservation, animal health, and human well-being.

## 6. Potential Risks to Human Health and Food Safety

Consumption of animal-origin foods contaminated with MPs and or NPs represents a growing concern for human health and food safety. Livestock and poultry are increasingly exposed to MPs/NPs through contaminated feed, water, inhalation, and environmental contact, leading to the accumulation of these particles in edible tissues and products such as meat, milk, and eggs, thereby creating a direct route of human exposure [22]. Several studies have reported MPs in chicken muscle, liver, and eggs, as well as cow’s milk. A study reported that microplastic contamination in chicken meat from the Middle East ranged from 0.03 ± 0.04 to 1.19 ± 0.72 particles per gram [106]. Another study reported that cow’s milk samples contained between 204 and 1004 microplastic particles (MPs) per 100 mL, with 69–89% of the particles having a surface area of ≤50 µm^2^, and reconstituted milk powders showed relatively higher MP levels than farm and liquid milk, predominantly composed of PE, PES, PP, PTFE, and PS polymers [107]. Once ingested, MPs/NPs can translocate across the human intestinal barrier and accumulate in organs such as the liver, kidneys, placenta, and brain [108,109]. Nanoplastics are potentially more dangerous than microplastics due to their small size, high reactivity, and ability to reach more remote locations and penetrate living cells [110]. Biological effects include oxidative stress, immune dysregulation, and disruption of cellular and genetic functions. In humans, chronic consumption of contaminated animal products may alter gut microbiota, impair gut barrier integrity, and facilitate systemic inflammation, along with other abnormalities reported in experimental models explained in other sections. The average adult is estimated to ingest 39,000–52,000 MP particles annually, with even higher exposure in plastic-polluted regions and meat-heavy diets [111].

**Figure 5:**
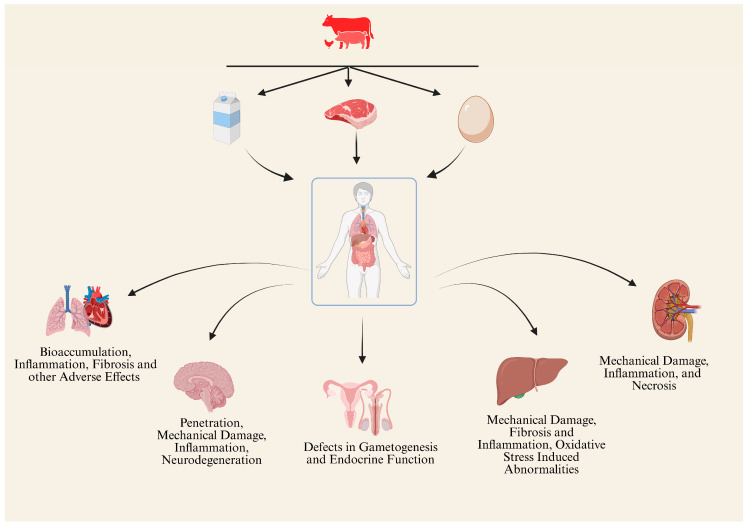
The pathway and effects of MPs-NPs on human health.

Furthermore, MPs/NPs often carry adsorbed toxicants like Pb, Ni, Co, and persistent organic pollutants, amplifying their toxicity [112,113]. Thus, regulating plastic exposure in animal farming and monitoring contamination in animal-derived foods are critical to protect public health and food security. The probable pathways and potential effects of MPs-NPs on human health have been demonstrated in Figure 5.

## 7. Research Gaps and Future Directions

Despite numerous reports on the detection of MPs in agricultural soil, marine sediments, water, and fish, there are no available reports on plastic particle contamination in animal feeds, terrestrial animals, or dried fish used in fish meal production. Additionally, no quantitative investigations have been conducted on NPs in either the environment or its inhabitants. Consequently, the current extent of MPs-NPs contamination in livestock and poultry products, including milk, meat, and egg in this region, remains unknown. Investigative studies are urgently needed to identify plastic particles in the food chain of these sources. Furthermore, the effects of plastic particles on milk and egg production, as well as on embryonic development, should be identified. Appropriate measures must also be taken to prevent the entry of plastic particles through these food animals, thereby safeguarding both animal welfare and public health.

## 8. Conclusions

This review highlights the emerging threat of MP-NP pollution to farm animals, with a specific focus on Bangladesh’s environmental context. The widespread presence of plastic particles in ecosystems, feed, air, and water sources presents considerable risks to animal health, affecting critical systems such as the reproductive, cardiovascular, nervous, immune, and digestive systems, while also contributing to genomic defects. The findings emphasize the urgent need for improved plastic waste management and pollution control strategies, particularly in overpopulated regions like Bangladesh, to mitigate the adverse effects of plastic pollution on agricultural systems, ensure the sustainability of livestock and poultry industries globally, and ultimately safeguard human health and the environment.

## Figures and Tables

**Table 1:** Reports on different microplastics isolated from livestock and poultry species.

Animal	Types of Microplastics	Amount Isolated	Organs Isolated From	Size of Microplastics	Reference
Cow	Polyvinyl Chloride, Polyethylene, Polymers of Styrene, and Polypropylene	PVC-P varies between 1.2 and 6.1 μg/g, Styr-P varies between 0.09 and 1.5 μg/g, and PE varies between 0.22 and 2.9 μg/g	Blood	≥700 nanometers	[54]
Cow	Nylon (polyamide)	0.14 items/g	Meat, Liver, and Tripe	<5 mm	[55]
Sheep	Fiber	0.13 items/g	Meat, Liver, and Tripe	<5 mm	[55]
Pig	Polymers of Styrene	PVC-P ranges from 1.7 to 17 μg/g, Styr-P ranges from 0.3 to 10 μg/g, and PE ranges from 2.1 to 33 μg/g	Blood	≥700 nanometers	[54]
Chicken	Not specified	Detected	Eggs	Not specified	[56]
Chicken	Polyvinyl Chloride, Low-Density Polyethylene, Polystyrene, and Polypropylene Homopolymer	Crop: 17.8 ± 12.1 particles/sample; Gizzard: 33.25 ± 17.8 particles/sample	Crop and Gizzard	50–500 µm	[19]

**Table 2:** Summary of investigations on the effects of microplastic exposure in various experimental models.

Experimental Models	Plastic Particles	Bioaccumulation	Effects	Reference
Mice	Polystyrene MPs	Heart, liver, spleen, lung, kidney, brain, large intestine, small intestine, uterus, ovary, and blood	Increased inflammation and oxidative stress in ovaries, reduced oocyte quality, and impaired reproductive outcomes.	[57]
Mice	Polystyrene MPs	No Information	Reduced sperm count and motility, increased deformity, decreased enzyme activity and testosterone levels, and induced oxidative stress.	[58]
Murine osteoblastic cell culture	Polystyrene NPs	Bone	Impact cell viability, induce oxidative stress and apoptosis, and alter gene expression related to inflammation and bone formation.	[59]
Mice	Polystyrene MPs	Organs of the digestive system and lower limb bones	Hematotoxicity and altered gene expression related to immune and metabolic processes.	[60]
Mice	Polystyrene MPs	No information	Delayed skeletal muscle regeneration, inhibiting myogenic differentiation, and promoting adipogenic differentiation.	[61]
Mice	Polystyrene MPs	Testis	Reproductive dysfunction, including decreased sperm quality, testosterone levels, testicular inflammation, and disruption of the blood–testis barrier.	[62]
Common fruit fly	Polystyrene MPs	Gut	Gut damage, shortened lifespan, disrupted sleep, reduced ovary size and egg-laying, and altered gene expression in various tissues.	[63]
Mice	Polystyrene MPs	Intestinal mucosa	Increased blood glucose and lipid levels, NAFLD activity, intestinal inflammation, and altered nutrient absorption.	[64]
Mice	Polyethylene MPs and NP	Gut	Induce gut macrophage activation and IL-1 signaling, leading to brain inflammation and cognitive decline in mice.	[65]
Mice	Polystyrene MPs	Gut	Gut microbiota imbalance, intestinal barrier dysfunction, and metabolic disorders in mice.	[66]
Mice	Polyethene MPs	No Information	Gut microbial changes, increased inflammation, and intestinal dysbiosis in mice.	[67]
Chicken	Polystyrene MPs	Liver	Significant liver damage through gut barrier disruption, microbial translocation, and tissue necrosis.	[68]
Mice	Polyethene NPs	Colon	Decreased colon mucin production, altered immune responses, and increased amino acid metabolism by changing colon microflora composition.	[69]
Lamb	Polystyrene MPs	No information	Decreased average daily gain, digestive disorders, gastrointestinal injury, and reduced meat quality.	[70]
Mice	Polystyrene NPs	Testes	Impaired sperm quality, disrupted testicular structures, and affected acrosome biogenesis in mice, with autophagy.	[71]
Mice	Polystyrene MPs and NPs	Organs of the digestive system, testis, lung, and bone marrow	Hematopoietic toxicity by disrupting bone marrow function, gut microbiota, metabolism, and inflammation.	[72]
Mice and their bone marrow cell culture	Polystyrene, polymethyl methacrylate, and polyethylene MPs	Heart, lung, kidney, spleen, organs of the digestive system, and bone marrow	Disrupt gut microbiota, impair hematopoietic stem cell function, and lead to their accumulation in multiple organs, including the gastrointestinal tract, liver, and bone marrow.	[73]
Mice	Polyethylene MPs	Stomach and spleen	Reduced body weight gain, altered immune responses, and negatively impacted reproductive and developmental outcomes in mice.	[74]
Chicken	No information	No information	Significantly impaired chicken growth performance and a notable imbalance in gut microbiota, altering microbial composition and structure.	[75]
Chicken	Polystyrene MPs	Muscle tissue	Affect metabolism, induce oxidative stress and neurotoxicity, alter metabolomic profiles, reduce meat quality, and impact muscle development by regulating neural function-related genes.	[76]

## Data Availability

The authors wish to disclose that this article includes no new data. All data referenced in this study are derived from the existing literature and publicly available sources. No additional data sets or materials are provided beyond those discussed in the article.
